# Incidence of Catheter-Associated Bloodstream Infections in Stem Cell Recipients—Should We Be “PICCy”?

**DOI:** 10.3390/cancers16061239

**Published:** 2024-03-21

**Authors:** Sławomir Milczarek, Piotr Kulig, Oliwia Piotrowska, Alina Zuchmańska, Ewa Wilk-Milczarek, Bogusław Machaliński

**Affiliations:** 1 Department of Hematology and Transplantology, Pomeranian Medical University, 71-252 Szczecin, Poland; oliw.piotrowska@gmail.com (O.P.); ewawilkmilczarek@gmail.com (E.W.-M.); 2 Department of General Pathology, Pomeranian Medical University, 70-111 Szczecin, Poland; piotrkulig@interia.eu (P.K.); zuchmanskaalina@gmail.com (A.Z.); 3 Pharmaceutical Facility of Pomeranian Medical University, Interfaculty Unit of Pomeranian Medical University, 71-899 Szczecin, Poland

**Keywords:** CLABSI, stem cell transplantation, PICC, CICC, MidLine catheter, central catheter, peripheral catheter

## Abstract

**Simple Summary:**

Proper vascular access is essential for stem cell transplantation (SCT). In the vast majority of transplantation centers, conventionally inserted central catchers (CICC) are the devices of choice. Although CICCs enable effective transplantation, their insertion is associated with life-threatening complications. Peripheral catheters (PCs) such as peripherally inserted central catheters (PICCs) and MidLine catheters (MLCs) appear to be suitable intravenous devices, yet are rarely used in this indication. We retrospectively appraised the infectious complications such as blood stream infection (BSI), febrile neutropenia (FN) and central line-associated bloodstream infection (CLBSI) in patients undergoing stem cell infusion through PC and conventionally inserted central catchers (CICCs), and evaluated their impact on transplantation outcomes. Our study showed that infection complications are independent of intravenous device and antibiotic prophylaxis. Considering that PCs are not associated with life-threatening complications, they should be considered for use more frequently in the stem cell transplantation setting.

**Abstract:**

The management of patients undergoing HSCT requires a multipurpose central venous catheter. Peripheral catheters (PCs), such as peripherally inserted central catheters (PICCs) and MidLine catheters (MLCs), appear to be adequate vascular catheters to be used for stem cell infusion, although their utilization in this indication is not yet common. We analyzed the infectious complications such as blood stream infection (BSI), febrile neutropenia (FN) and central line-associated bloodstream infection (CLBSI) in patients undergoing stem cell infusion through PC and conventionally inserted central catchers (CICCs), and evaluated their impacts on transplantation outcomes. Our results reveal no statistically significant differences between different types of catheter in terms of FN, BSI and CLABSI. Moreover, transplantation outcomes were comparable between the groups. Interestingly, according to our data, there were no differences in terms of abovementioned infectious complications between individuals who received antibiotic prophylaxis and those who did not. Our study has shown that infection complications are independent of the intravenous device and antibiotic prophylaxis. Considering that PCs are not associated with life-threatening complications, they should be considered more frequently in the stem cell transplantation setting.

## 1. Introduction

Hematopoietic stem cell transplantation (HSCT) is the standard of care for hematological malignancies and non-malignant disorders [1]. The implantation of a multipurpose venous catheter, due to its complex nature, is mandatory for successful HSCT. Such catheters enable, among other things, parenteral drug administration [2]. Typically, the two types of catheters used for implantation include conventionally inserted central venous catheters (CICCs), inserted through large central veins, and peripherally inserted central catheters (PICCs), implanted through peripheral veins, usually of the upper limb [3]. CICCs in 1–19% of cases are associated with significant complications during implantation [4]. On the contrary, implanting PICCs is safe and lacks many complications due to the location of the initial puncture site [5,6,7].

PICCs are inserted in the non-dominant upper arm under ultrasound guidance and terminate in the cavo-atrial junction [8]. The basilic vein is the best insertion location, and the arm’s medial distal part is the recommended puncture site. The catheter/vein ratio should not exceed 45%, as advocated by the Infusion Therapy Standards of Practice [9]. An alternative subtype of PICCs is MidLine catheters (MLCs). MLCs are devices inserted into the peripheral veins of the upper extremity and terminate in the peripheral veins, not the central veins [10]. More precisely, the tip of the MLC catheter should be located at or below the axillary vein, distal to the shoulder [11]. Both catheter types facilitate the administration of chemotherapy, antibiotics, parenteral nutrition, blood products, and systematic sample collection.

Regardless of the catheter type, an indwelling vascular line increases the risk of central line-associated bloodstream infections (CLABSI) [12,13]. The mortality rate associated with CLABSI varies from 12% to 40%, depending on factors such as patient comorbidities, catheter type, and the type of microorganism causing the infection [14]. Current data indicate CLABSIs are estimated to occur in cancer patients at a frequency of 0.5 to 10 cases per 1000 CICC-days [15]. The CLABSI incidence rate is higher in hematologic patients, reaching 17.3 in aggressive hematological malignancies and 21% during the pre-engraftment phase in patients undergoing HSCT [16,17]. Several retrospective studies have concluded that peripherally implanted devices could alleviate the incidence of CLABSI among patients suffering from hematological malignancies [5]. Additionally, the information on the safety and usefulness of different peripheral catheters among stem cell recipients is scarce. Previously, we have demonstrated that prolonged PBSC infusion through PC does not affect engraftment kinetics, and therefore transplantation outcomes [18]. We also demonstrated that the incidence of catheter-related thrombosis did not differ between CICC and PC [19].

This retrospective study presents the results of a comparative analysis of catheter-related bloodstream infections in patients undergoing stem cell transplantation with the use of alternative vascular devices, including central and midline catheters. The primary objective was to evaluate the incidence of CLABSI associated with PICCs and MLCs in comparison to CICCs.

## 2. Materials and Methods

### 2.1. Study Design

#### 2.1.1. Patients

From January 2021 to March 2023, we investigated 86 consecutive autologous and allogeneic peripheral blood stem cell transplantation (PBSCT) procedures at the University Hospital No. 1 in Szczecin (Poland). All enrolled patients signed informed consent. The study was conducted in accordance with the Declaration of Helsinki. The study was approved by the Bioethical Committee of Pomeranian Medical University in Szczecin (RPW/10177/2022P). Patient and catheter characteristics are presented in Table 1 in the Results section.

The patients were divided into two groups—the study group consisted of 41 patients who underwent transplantation with peripheral catheters (PCs), both PICCs and MLCs, while the control group consisted of 45 patients with inserted CICC. Most patients undergoing ASCT preceded by melphalan conditioning had MLCs inserted, as melphalan has a pH of 6, and therefore can be administered to large peripheral veins. During the procedure, the catheters were used as the multipotential vascular access, facilitating high-dose chemotherapy, stem cell infusion, large-volume fluid therapy, immunosuppressive treatment, anti-infective agents, blood-derived products infusion, and parenteral nutrition.

Until January 2022 we routinely used CICC for every transplant procedure, both autologous and allogeneic. Our unit initiated the peripheral catheter program for PICC and MLC in January 2022. Since then, we have routinely used them for stem cell transplantations unless contraindications occur. The most common contraindications for their implantation involve poor peripheral vasculature with the high catheter-to-vein ratio for small-size catheters and an expected complicated course of transplantation (e.g., AML not in remission, sequential conditioning). Based on our experience, when a severe course of transplantation requires a simultaneous infusion of vasopressor drugs, parenteral nutrition, intravenous immunosuppression, antibiotics, blood products, and supportive drugs, CICC is a better option than PICC, predominantly because CICC has a larger diameter and more lumens than PICC. For these patients, we typically implant CICC from the outset. However, if the situation unexpectedly develops, we may implant an additional MLC to a preexisting PICC.

#### 2.1.2. Technical Aspects of Insertion Procedure and Catheter Characteristics

Central lines and peripheral catheters were implanted and supervised at our center by the “Vascular Access Team” under ultrasonographic control.

All peripheral devices used in our unit were deprived of valves. The explanation for this practice is that valveless catheters allow a faster stem cell infusion rate, which, as demonstrated in our previous study, is already significantly decreased if performed by PICC. Additionally, central venous pressure measurement is more feasible if the catheter is valve-free. While PICC and MLC were typically implanted into the brachial vein unless there were certain anatomical variants incompatible with successful procedure, CICCs were inserted into the right subclavian vein. Thereafter, each patient was routinely referred to chest X-ray. No radiographs were performed after MLC placement as it is not routinely recommended due to shorter lengths—typically, they do not exceed the axillary vein.

Most PICCs were 5 Fr, and nine patients had different catheter sizes: six 4 Fr and three 6 Fr. Regarding MLC, nine patients had 4 Fr and eight had 5 Fr. Most peripheral catheters were dual-lumen, except for three triple-lumen PICCs, five single-lumen PICCs, and two single-lumen MLCs. The size of the PICC and MLC depended on the diameter of the vein and the catheter/vein diameter ratio. The acceptable ratio was 0.33–0.45 at the site of venipuncture and onward. A number of lumens were dependent on assumed medical needs during treatment, but most catheters were double-lumen devices.

#### 2.1.3. Management of Vascular Line

Besides the tailored approach to choosing a peripheral catheter size and the number of lumens, we routinely administered low-molecular-weight heparin (LMWH) from the day of insertion until the platelet count decreased <30 G/L. The heparin was re-administered once the patient had been engrafted and platelets exceeded >30 G/L. The manufacturer does not routinely recommend this practice. Still, our experience suggests that this practice further decreases the risk of CRT. Patients in the CICC group had thromboprophylaxis with LMWH due to other factors. The rest had no prophylaxis. All unit nurses are trained in managing both peripheral and central lines. Routine pulsatile flushing with 0.9% saline was performed before and after every use. The exit site was examined visually daily for signs of inflammation or infection. The limb was additionally assessed for symptoms of collateral vein enlargement suggestive of DVT. The dressing was routinely changed every 72 h or if needed. If febrile neutropenia occurred, we routinely obtained blood samples from the peripheral veins of both upper extremities and all lumens of the central catheter. If blood cultures were positive, time-to-positivity was regularly assessed. The reasons for catheter removal were completion of therapy, catheter infection, or a mechanical complication.

#### 2.1.4. Antibiotic Prophylaxis

Since the inception of our Bone Marrow Transplantation Department in 2018 until September 2022, fluoroquinolones (FQ) have been the cornerstone of our prophylactic regimen during the neutropenic phase until the engraftment of neutrophils. However, with periodic yearly epidemiologic evaluations, we have detected an increasingly worrisome trend of FQ resistance among local Encetobacteriacae at our institution. Therefore, a strategic shift to cephalosporins has been implemented as a prophylactic treatment. Specifically, ceftriaxone has been identified as the most effective agent against Gram-negative pathogens and has thus become the new standard of care in our unit.

#### 2.1.5. Statistical Analysis

Continuous variables are expressed as mean (median) and interquartile range (IQR). The Shapiro–Wilk test was implemented to assess the distribution of continuous variables. As constant variables were not normally distributed, the Mann–Whitney U test was implemented to compare the differences between the groups. The Fisher exact test was used to assess differences in the categorical variables between the groups. When relevant, post hoc pairwise comparison with Bonferroni correction for multiple testing was implemented. A logistic regression model was used to determine if a catheter type predicts infection complications. *p* value < 0.05 was considered statistically significant. All calculations were performed in RStudio.

### 2.2. Definitions

A. CLABSI—CLABSI was considered as a primary infection if there were no other clinical signs or symptoms for another infectious focus. Centers for Disease Control and Prevention (CDC) criteria (National Healthcare Safety Network) for primary (laboratory-confirmed) BSI were applied. The catheter association of the infection was defined as follows: (i) the catheter was in place for at least 48 h prior to onset of sepsis, and/or (ii) there was microbiologic growth (bacteria and/or fungi) of at least 15 colony-forming units (CFU) on the catheter tip identical to a positive blood culture sample, and/or (iii) the difference in time to positivity between a central and a peripheral drawn blood culture was more than 2 h.
B. BSI (bloodstream infection)—(definition consistent with CDC criteria [20]) BSI must meet at least 1 of the following criteria:
  i. The patient has a recognized pathogen cultured from 1 or more blood cultures, and the organism cultured from blood is not related to an infection at another site;
  ii. The patient has at least 1 of the following signs or symptoms—fever (>38 °C), chills, or hypotension, and signs and symptoms and positive laboratory results are not related to an infection at another site

## 3. Results

### 3.1. Characteristic of the Study Group

Overall, 41 PCs and 45 CICCs were inserted into 86 patients. The demographic characteristics, underlying hematologic diseases, and procedure outcomes are listed in Table 1. Among the PC group, 24 individuals underwent HSCT using PICCs, while 17 used MLCs. Our cohort did not differ in age or sex. Most of the procedures were autologous transplantations (*n* = 62) but allogeneic transplantations were also included (*n* = 24). There was a significantly increased length of stay in the CICC group, most likely because this was the dominating vascular access in allogeneic transplantations.

### 3.2. Comparison between the Catheters

Table 2 presents a comparison between CICCs and PCs. The incidence of CLABSI, FN, and catheter-unrelated BSI was analyzed. FN occurred in 60% and 63.4% of patients with CICCs and PCs, respectively. Catheter-unrelated BSI occurred in 20% and 21.95% of patients with CICCs and PCs, respectively. Similarly, 13.33% in CICC and 12.2% in the PC group succumbed to CLABSI. There were no statistically significant differences between the groups.

Next, to determine the differences in infectious complications between different types of PCs and CICCs, the PC group was divided into two subgroups—MLCs and PICCs. The CLABSI, FN, and BSI proportions do not differ from those in Table 2. The CLABSI and BSI in MLC patients had the same incidence—11.76%, while FN was diagnosed in 58.82% of MLC individuals. CLABSI was diagnosed in 12.5% of PICC patients, BSI in 12.5% and FN in 66.67% of PICC cases. Although FN was the most prevalent among the PICC group (66.67%), the analysis revealed no differences between all three types of catheter. The results are presented in Table 3.

Logistic regression was used to analyze if a catheter type predicts infectious complications (Figure S1). Similarly, the analysis detected no relationship between catheter type and infectious complications, i.e., CLABSI, Febrile neutropenia and BSI.

Table 4 presents the relationship between antibiotic prophylaxis and infectious complications; 70.93% of patients in our cohort received antibiotic prophylaxis, and there was no difference between the CVC and PC groups (Table 1). Moreover, patients who received prophylaxis did not differ from those who did not in terms of CLABSI, FN, BSI, and infection (defined as CLABSI or BSI).

## 4. Discussion

The use of central vascular access in transplant patients has improved clinical management, allowing intensive and supportive treatments to be administered. However, the use of CICCs continues to be associated with early (hemo/pneumothorax) and delayed (CLABSI, catheter-related thrombosis) complications. PICC lines offer dependable central venous access for various patient categories, mainly due to the ease of the insertion technique, which allows for possible bedside placement and a low risk of complications. The systematic meta-analysis by Chopra et al. showed that PICCs are associated with a lower risk of CLABSI in an outpatient setting and a comparable risk of CLABSI to CICCs in hospitalized patients [21]. Interestingly, an extensive comparative analysis by Nakaya et al. confirmed a lower incidence of CLABSI in patients with PICCs than in CICCs, and revealed a microbiological shift and decrease in Gram-positive cocci and an increase in Gram-positive bacilli (*p* = 0.001) responsible for CLABSI among patients with PICCs [22]. Detailed data regarding PICC-related infections in transplant recipients are mostly retrospective and scarce, suggesting a CLABSI incidence ranging from 3 to 40% [6,23,24] with significantly higher incidence among allogeneic HSCT, probably due to prolonged neutropenia and profound immunodeficiency. It is worth emphasizing that the incidence of CLABSI, even among central catheters, varies depending on the site of vascular catheter implantation. According to Heidenreich et al., among hematological patients receiving intensive induction or high-dose therapy, CLABSI occurred significantly more often when neutropenia exceeded 6 days (*p* = 0.024) [25], or the catheter was inserted into the internal jugular vein (IJV) compared to the subclavian vein probably due to the presence of facial hair, neck movements, and worse dressing adhesion [26]. A study performed by Snarski et al. on behalf of the Infectious Diseases Working Party of The European Society for Blood and Marrow Transplantation (IDWP EBMT) compared the risks of catheter-related infectious complications in two sites of catheter insertion (IJV and SCV) among allogeneic stem cell recipients [27]. The analysis of 232 patients indicated a statistically significant difference favoring the subclavian approach (23% IJV vs. 13% SCV (OR 2.03 (1.01–4.06), *p*  =  0.047)). In accordance with the presented data, in our center, when CICCs are inserted, we favor the subclavian approach to decrease infectious complications further, and increase the patients’ comfort.

Despite the emerging data suggestive of low CLABSI incidence in hematology patients treated with PICCs, peripheral access is rarely the physician’s first choice. According to our knowledge, this study is the first specifically conducted to compare the incidence of catheter-related infections in adult patients undergoing stem cell transplantation with different catheter types.

The incidence of febrile neutropenia in our study was 60% and 63.4% in patients with CICCs and PCs, respectively, which corresponds to previously reported data on FN occurrence in transplantation setting [28]. The FN incidence was independent of antibiotic prophylaxis, as depicted in Table 4.

Our study reports the lower-than-expected incidence of endogenous/unknown bloodstream infections in stem cell recipients, reaching 20% and 21.95% for CICCs and PCs, respectively. There were no statistically significant differences between either group with alternative peripheral catheters. Furthermore, antibiotic prophylaxis had no impact on the incidence of bloodstream infections in general and in either type (BSI/CLABSI). Lately, published data detailing the incidence and epidemiology of BSI in HSCT patients have shifted during the last few decades. A large Spanish retrospective study by Puerta-Alcalde et al. reported the decreased occurrence of BSI over years. They analyzed 1164 BSI episodes in transplant recipients (71.6% allogenic and 29% autologous) [29].

Finally, the incidence of CLABSI in our group was also low—13.33% in CICC and 12.2% in PC, respectively. Among PC subgroups, CLABSI occurred in 11.76% of MLC and in 12.5% of PICC patients, which is also lower than expected. The differences between the catheters were not statistically significant. Due to a low number of infections, we were unable to assess the causative pathogens. Interestingly, fluoroquinolone (FQ) prophylaxis did not significantly impact CLABSI incidence in either group despite prolonged time to neutrophil recovery in the non-prophylaxis group. The type of infection (FN/BSI/CLABSI) also had no impact on the transplantation procedure outcome. None of the patients died of infectious complications.

To sum up, PCs are associated with less severe mechanical complications and represent a less invasive approach. Moreover, despite many concerns, prolonged PBSC infusion through PC does not affect transplantation outcomes [18], and PC is not a risk factor for CRT in the stem cell transplantation setting [19]. We believe most autologous transplantations could be performed with MLC or PICC instead of CICC. Allogeneic transplantation requires a more tailored approach and detailed patient and treatment factor analysis for choosing the proper vascular access. In our transplantation unit, PICCs are routinely used in reduced-intensity conditioning allo-HSCT. Further prospective studies and appropriate statistical evaluations are needed to establish the role of PICCs and MLCs in preventing infections in stem cell recipients.

Another interesting finding is the lack of effectiveness of FQ prophylaxis in our study. Due to increasing FQ resistance, and changes in the microbiome caused by FQ, we hypothesize that the prophylactic strategy needs to be re-evaluated, to determine whether it could be omitted and replaced by an aggressive microbiological approach with PCR and T2 rapid assessments.

We acknowledge that the retrospective character of this study has a few limitations. First, due to a low number of allogeneic recipients, we were unable to compare both types of transplantations properly. Second, we did not take the disease status into account, which could potentially alter the incidence of CLABSI, but only one patient in the allogeneic group was transplanted in progressive AML, while all other patients were in remission. However, adjustment for the other covariates (age, sex, antibiotic prophylaxis) could decrease the bias between CICC and PICC groups to an acceptable degree. Third, this study did not include the mucosal barrier injury with laboratory-confirmed bloodstream infection (MBI-LCBI) criterion. Therefore, the number of CLABSI events may have been overestimated [30].

## 5. Conclusions

In conclusion, our study has demonstrated that the use of peripheral vascular catheters does not lead to an increased incidence of central line-associated bloodstream infections (CLABSI), regardless of the type of catheter used. Therefore, we recommend that peripherally inserted central catheters (PICC) and midline catheters (MLC) be considered as the primary devices, unless there are specific clinical justifications that necessitate the use of a more invasive approach, such as suspected complex transplantation procedures that require multiple continuous infusions of physically incompatible drugs or parenteral nutrition. Our findings underscore the importance of carefully considering the risks and benefits associated with different types of catheters, and tailoring their use to the specific needs of individual patients.

## Figures and Tables

**Table 1:** Patient and catheter characteristics. AML—acute myeloid leukemia, MDS–myelodysplastic syndrome, ALL–acute lymphoblastic leukemia, CMML–chronic myelomonocytic leukemia, PCM—plasma cell myeloma, GCT—germ cell tumor, HL—Hodgkin lymphoma, MCL–mantle cell lymphoma, DLBCL—diffuse large B-cell lymphoma, BPDCN—blastic plasmacytoid dendritic cell neoplasm, PTCL—peripheral T-cell lymphoma, Allo-HSCT—allogeneic stem cell transplantation, Auto-HSCT—autologous stem cell transplantation, MEL—melphalan, CE—carboplatin/etoposide, FluBu4—fludarabine/busulfan, BuMel—busulfan/melphalan, FluTBI—fludarabine/total body irradiation, BeEAM—bendamustine, etoposide, cytarabine, melphalan, Fr—French gauge (1 Fr = 1/3 mm), CLABSI—Central Line-Associated Bloodstream Infection. *p* value was calculated where applicable. N—number, IQR—interquartile range. Statistically significant results are shown in bold. *p* value was calculated when applicable.

	Peripheral Catheter; N/Mean (Median); IQR	Central Catheter; N/Mean (Median); IQR	*p*-Value
Age	55.44(57); 45–66	53.96(60); 44–66	0.70
Sex F/M	18/23	16/29	0.51
**Diagnosis**			
AML	5	8	N/A
MDS/AML	1	0	N/A
ALL	0	2	N/A
CMML	1	0	N/A
GCT	8	2	N/A
PCM	24	14	N/A
HL	0	9	N/A
MCL	1	2	N/A
DLBCL	1	5	N/A
BPDCN	0	1	N/A
PTCL	0	2	N/A
**Type of HSCT**			
auto-HSCT	32	30	0.34
alloHSCT	9	15	
**Length of stay (days)**	24.27(22); 21–27.25	30.67(29); 22–33	0.007
**Neutrophil engraftment (days)**	12.37(11); 11–12	13.82(12); 11–14	0.19
**Platelet engraftment (days)**	14.34(13); 12–15	16.09(14); 12–17	0.16
**Catheter type**			
PICC	24	N/A	N/A
MLC	17	N/A	N/A
CICC	N/A	45	N/A
**Catheter size**			
Fr4	15	0	N/A
Fr5	23	0	N/A
Fr6	3	0	N/A
Fr7	0	45	N/A
**Number of lumens**			
1	7	0	N/A
2	31	0	N/A
3	3	45	N/A
**Insertion site**			
Right subclavian vein	0	37	N/A
Left subclavian vein	0	5	N/A
Right jugular vein	0	3	N/A
Left basilic vein	28	0	N/A
Right basilic vein	10	0	N/A
Left brachial vein	1	0	N/A
Right brachial vein	2	0	N/A
**Duration of neutropenia (days)**	7.78(8); 6–10	8.84(9); 7–10.25	0.13
**Prophylaxis Yes/No**	26/15	35/10	0.16

**Table 2:** Comparison between patients with CICC and PC. Fisher exact test; *p* < 0.05 is considered statistically significant. PC—peripheral vein catheter; BSI—bloodstream infection; CLABSI—Central Line-Associated Bloodstream Infection; CICC—conventionally inserted central catheter; PICC—peripherally inserted central catheter; MLC—MidLine catheter; PC—peripheral catheter.

Infection	CICC	PC (PICC or MLC)	OR, 95% CI	*p*-Value
	**YES/NO**	**YES/NO**		
CLABSI	6/39	5/36	0.90, 0.20–3.90	1
FN	27/18	26/15	1.15, 0.44–3.03	0.82
BSI	9/36	9/32	1.12, 0.35–3.64	1

**Table 3:** Comparison between patients with PC, MLC, and PICC. Fisher exact test followed by post hoc pairwise comparison with Bonferroni correction for multiple testing; *p* < 0.05 is considered statistically significant. BSI—bloodstream infection; CLABSI—Central Line-Associated Bloodstream Infection; CICC—conventionally inserted central catheter; PICC—peripherally inserted central catheter; MLC—MidLine catheter; PC—peripheral catheter.

Infection	Catheter Type	NO	YES	*p*-Value
**CLABSI**	CICC	39	6	1
	MLC	15	2	
	PICC	21	3	
**BSI**	CICC	36	9	0.6
	MLC	15	2	
	PICC	21	3	
**FN**	CICC	18	27	0.84
	MLC	**7**	10	
	PICC	**8**	16	

**Table 4:** The relationship between antibiotic prophylaxis and infectious complications; 70.93% of patients in our cohort received antibiotic prophylaxis, and there was no difference between the CVC and PC groups (Table 1). Moreover, patients who received prophylaxis did not differ from those who did not in terms of CLABSI, FN, BSI, and infection (defined as CLABSI or BSI).

	Prophylaxis	No Prophylaxis	*p* Value
**Infection type**	YES/NO; mean(median); IGR	YES/NO; mean(median); IGR	
CLABSI	8/53	3/22	1
FN	37/24	16/9	0.81
BSI	12/49	6/19	0.77
Infection (CLABSI or BSI)	18/43	9/16	0.61
Catheter type (CICC/PC)	35/26	10/15	0.16
Neutrophil engraftment	12.66(12); 11–13	14.28(12); 11–16	**0.02**
Platelet engraftment	15.18(14); 12–16	15.44(13); 12–15	0.68
Duration of neutropenia	8.1(8); 6–10	8.88(9); 7–11	0.23
Length of hospitalization	27.75(25); 20.75–31.25	27.44(25); 22–32	0.64

## Data Availability

Data are available upon request from the correspondence authors.

## Supplementary Materials

The following supporting information can be downloaded at: https://www.mdpi.com/article/10.3390/cancers16061239/s1, Figure S1: catheter type predicts infectious complications; Table S1: Causative pathogens; Table S2: Time (in days) from catheter implantation to infection diagnosis; Table S3: Time (in days) from the beginning of neutropenia to infection diagnosis.
