# Supplementary material for: Incidence of Catheter-Associated Bloodstream Infections in Stem Cell Recipients—Should We Be “PICCy”?
Source: Cancers (Basel). 2024 Mar 21;16(6):1239. doi: 10.3390/cancers16061239 (PMC10969275; doi:10.3390/cancers16061239)
Supplement: Supplementary file 1 [file cancers-16-01239-s001.zip › Supplementary file 1.pdf]

Causative pathogen	Peripheral catheter		Central catheter	
	BSI	CLABSI	BSI	CLABSI
Escherichia coli	3	1	1	1
Escherichia coli ESBL			4	
Staphylococcus aureus	1			
Klebsiella pneumoniae ESBL	1	1		1
Staphylococcus mitis	1			
Klebsiella variicola	1			
Staphylococcus epidermidis MRCNS	2			2
Staphylococcus hominis	1	1		
Enterococcus faecium		1	2	
Streptococcus vestibularis		1		
Enterococcus faecalis			1	
Enterobacter cloacae ESBL			1	
Streptococcus oralis			1	
S.viridans			1	
Staphylococcus epidermidis				2
Staphylococcus haemolyticus				1

Table S1. Causative pathogens.

Infection	Central catheter	Peripheral catheter	P value
FN	15.48(17),12.50-18	16.92(12.5), 11-18.25	0.27
BSI	25.44(19), 18-23	14.11(14), 11-14	0.01
CLABSI	18(15), 14-18.25	11.4(11), 11-12	0.04

Table S2. Time (in days) from catheter implantation to infection diagnosis. Data are reported as mean(median) and interquartile range. Mann Whitney U test, $p < 0.05$ is considered statistically significant. FN – febrile neutropenia; BSI – bloodstream infection; CLABSI – Central line-associated bloodstream infections

Infection	Central catheter	Peripheral catheter	P value
FN	6.78(6),5-8.5	7.192(5), 4-7	0.43
BSI	12.33(9), 5-13	7.78(6), 5-7	0.45
CLABSI	13.5(10), 7.5-13.25	4.2(5), 1-6	0.03

Table S3. Time (in days) from the beginning of neutropenia to infection diagnosis. Data are reported as mean (median) and interquartile range. Mann Whitney U test, $p < 0.05$ is considered statistically significant. FN – febrile neutropenia; BSI – bloodstream infection; CLABSI – Central line-associated bloodstream infections.
